# Abscopal Effect in Metastatic Melanoma: Generating Clinical Insights From Radiation-Induced Immune Response

**DOI:** 10.7759/cureus.72853

**Published:** 2024-11-01

**Authors:** David M Nguyen, Baovy N Phan, Sunil A Reddy

**Affiliations:** 1 Oncology, Sutter Medical Group of the Redwoods, Santa Rosa, USA; 2 Biology, Union College, Schenectady, USA; 3 Oncology, Stanford University Medical Center, Stanford, USA

**Keywords:** abscopal, immunotherapy, malignant, melanoma, metastasis

## Abstract

Despite modern advancements in systemic therapies, melanoma remains a highly malignant cancer, with persistent resistance to therapies such as checkpoint inhibitors and inhibitors of mutated BRAF V600E. Current therapy for targeting metastatic melanoma remains palliative radiation therapy, particularly directly at symptomatic sites. Extraordinarily, few case studies have reported locally targeted radiation resulting in regression of distal non-targeted lesions. This rare phenomenon was coined as the abscopal effect and presents with increasing frequency due to the advancement of systemic therapy. This narrative review attempts to understand the underlying mechanisms behind the abscopal effect through clinical data from 21 melanoma patients reported in 19 case studies. Case identification focused on patients who had progressed on systemic therapy before receiving radiation. Our study observed a mean total Gy of 34 (median total 30 Gy) and mean fractionation Gy of 8 (median fractionation 7.5 Gy) with increased frequency of reported abscopal effects with incorporation of modern systemic immunotherapies. The reviewed cases suggest that combining radiation with immunotherapy may enhance systemic tumor control, though further research is required to better understand the underlying mechanisms and improve treatment outcomes.

## Introduction and background

Malignant melanoma is considered a highly immunogenic tumor, characterized by a high mutation burden compiled with an abundance of neoantigens, which makes it particularly responsive to immunotherapies. However, it continues to be a highly aggressive malignancy despite advances in systemic therapy. It ranks as the fifth most common cancer, with an estimated 100,350 new cases and 6,850 deaths in 2020, despite the use of modern systemic therapy such as checkpoint inhibitors and inhibitors of mutated BRAF V600E [1]. Prior to the arrival of these therapies, there was no treatment with a consistent overall survival benefit. The prognosis was somber, with overall survival being less than one year and five-year overall survival <10% in metastatic melanoma [2]. Modern immunotherapy has produced durable responses, with 52% of a subset of patients remaining alive at five years [3]. Unfortunately, the prognosis continues to be poor due to limited options after resistance or progression on systemic immunotherapy and/or targeted BRAF therapy. This remains a major unmet need.

Palliative radiation is used to treat metastatic melanoma, especially at symptomatic sites, to provide relief. It has been observed that locally targeted radiation may promote regression of distant, non-targeted lesions. This was first reported in 1953 and is termed the abscopal effect [4]. Given its rare occurrence, it has been regarded more as an oddity than a reliable phenomenon. With the advancement of systemic therapy, including immune checkpoint blockade and BRAF inhibitors, the abscopal effect in melanoma is being increasingly reported [5].

The abscopal response has been described in immunogenic tumors such as melanoma. In melanoma, lesion infiltration by T lymphocytes is associated with a better clinical prognosis [6]. While the exact biological mechanism of the abscopal effect is not completely understood, it may be mediated by immune activation through immunotherapy and radiation [7]. Due to rarity, it's unclear if there are any patient-specific factors that are associated with abscopal responses.

This narrative review attempts to gather clinical insights from a collection of published melanoma patients in whom the abscopal phenomenon has been reported.

## Review

Materials and methods

A search of the literature was conducted through PubMed and Google Scholar using the keywords “abscopal”, “immunotherapy”, “malignant”, “melanoma”, and “metastasis. Publications, including case reports, case series, letters to editors, clinical trials, and retrospective series were considered. References of accepted publications were also assessed for additional case data and analysis. Primary clinical data with regards to the age of abscopal, sex, location of cutaneous melanoma, stage, adjuvant systemic, adjuvant radiation, distant metastatic site, disease status prior to radiation, radiation sites and dose, subsequent results after radiation, overall survival in months, abscopal effect in months, duration to progression, and status of the patient at the time of publication were categorized for analysis and interpretation. While it’s felt that the radiation abscopal effect can occur before, during, or after systemic therapy, it is difficult to ascertain if distal responses were driven by systemic therapy or the abscopal effect. Therefore, we have chosen studies that progressed on systemic therapy since this reflects real-world practices where palliative xrt is used for symptom management after a patient has started systemic therapy. To improve the quality of data, publications were excluded for the following: (1) if systemic treatment with RT was given at the same time or systemic therapy was given after RT, confounding the ability to assess the cause of response, (2) if the status of patient (alive/dead) was not reported, (3) if abscopal sites could not be confidently attributed to malignancy (not previously sampled) since radiographic inflammation/infection can present in similar ways and subsequently resolve confounding interpretation, and 4) less than 75% of clinical parameters were reported per case (Table 1). Non-English articles were translated using Google Translate (two studies; Google LLC, Mountain View, CA, USA). M1 staging was defined retrospectively via Melanoma AJCC 8th Edition. Duration of the abscopal effect was defined by the time of radiation therapy to the last contact or subsequent progression. Overall survival was from the date of diagnosis to reported death or last contact. The primary data was organized into a database for which subsequent statistical techniques were applied using WEKA (WekaIO, Inc., Campbell, CA, USA).

**Table 1:** List of exclusion criteria

List of exclusion criteria	
1	Systemic therapy was given concurrently or post-radiotherapy before imaging confirmation of the abscopal effect
2	Patient status not reported (alive/dead)
3	Abscopal sites not confidently attributed to malignancy
4	Less than 75% of clinical parameters reported

Results

Nineteen publications were included in our study (Table 2), resulting in 21 patients available for analysis. The mean age was 66 and ranged from 28-95 years and affected both genders (11 men and 8 women; unknown 2).

**Table 2:** Publications and selected notes

Study number (#)	Reference	Pertinent notes (systemic, radiated location)
1	[8] Kingsley, 1975	None, groin-extremity
2	[9] Postow et al., 2012	Ipilimumab, a pleural-based paraspinal
3	[10] Stamell et al., 2013	None, head and neck
4	[11] Sullivan et al., 2013	Vemurafenib, brain
5	[12] de la Cruz et al., 2014	None, brain
6	[13] Okwan-Duodu et al., 2015	Interleukin-2, brain
7	[14] Thallinger et al., 2015	Ipilimumab, brain
8	[15] Komori et al., 2018	Nivolumab, skin-back
9	[16] Roger et al., 2018	Nivolumab, iliac-lymph node
10	[17] Sims-Mourtada et al., 2018	Pembrolizumab, lung
11	[18] Galkin et al., 2018	None, brain
12	[19] Tsui et al., 2018	Pembrolizumab, brain
13	[20] Gutkin et al., 2018	Ipilimumab, liver
14	[21] Moran et al., 2019	Nivolumab and ipilimumab, lung
15	[22] Anderson and Arcaro, 2019	Patient 3: ipilimumab, head and neck; patient 4: none, extremity
16	[23] Trommer et al., 2019	Patient 1: pembrolizumab, brain; patient 2: pembrolizumab, brain
17	[24] Silva et al., 2019	None, groin-extremity
18	[25] Watanabe et al., 2020	Patient 2: pembrolizumab, liver
19	[26] Igarashi et al., 2020	Nivolumab, brain

As shown in Table 3, the skin's primary location varied (5 in the head and neck, 6 in the trunk, and 8 in the extremity; unknown 2).

**Table 3:** Baseline demographic of patients

Characteristic	Value
Number of patients, n	20
Mean age in years, n	66 (71 alive vs. 49 dead)
Sex	
Male	11
Female	8
Unknown	2
Skin primary site	
Head and neck, n (%)	5 (24)
Trunk, n (%)	6 (29)
Extremity, n (%)	8 (38)
Unknown, n (%)	2 (9)
Stage	
I, n (%)	1 (5)
II, n (%)	3 (14)
III, n (%)	10 (48)
IV, n (%)	3 (14)
Unknown, n (%)	4 (19)

All patients had undergone prior standard treatments (Table 4). Sixteen patients had prior surgery for resection of the primary site of metastasis. Only five patients had adjuvant radiation, and the majority (16 patients) had no adjuvant systemic therapy. The median time to recurrence after surgery was 48 months.

**Table 4:** Key abscopal features (at metastatic or recurrence)

Characteristic	Value
Systemic therapy	
Immunotherapy, n (%)	14 (66)
BRAF, n (%)	1 (5)
None, n (%)	6 (29)
# of prior lines	
0 patients	17
1 patient	3
2 patients	1
M stage	
M1a (%)	3 (14)
M1b (%)	5 (24)
M1c (%)	5 (24)
M1d (%)	8 (38)
Mean time on systemic therapy prior to radiation (months)	4.7
Progression, stable, PR, CR n (%)	
Progression	20 (100)
Stable	0 (0)
Partial response	0 (0)
Complete response	0 (0)
Radiation therapy	
Total Gy: mean (alive vs. dead)	34 (36 vs. 28)
Total Gy: median	30
Fractionation Gy: mean (alive vs. dead)	8 (10 vs. 2)
Fractionation Gy: median	8
Radiation to lymph node	Yes: 7
Radiation to lymph node	No: 14
Local radiation response	
Progression	0
Stable	0
Partial response	10
Complete response	11
Abscopal response	
Progression	0
Stable	0
Partial response	8
Complete response	13
Average abscopal response duration (months) (alive vs. dead)	19 (21 vs. 14)
Average overall survival (months) (alive vs. dead)	78 (90 vs. 34)

In terms of key abscopal features (Table 5), 100% had progression prior to radiation. The total radiation mean dose was 34 Gy (range 14-60), and the median dose was 30 Gy. The mean and median dose per fraction were found to be 8 Gy (range 1.2-20 Gy). Only seven had radiation delivered to lymph nodes, while 14 did not. There was an average of 4.7 months of systemic therapy prior to the use of radiation. Once the abscopal effect was achieved, a mean time of 19 months passed before disease progression or follow-up was last reported (range 2.5 to 78 months). Mean overall survival was 78 months (range 13 to 239 months).

**Table 5:** Initial treatment characteristics of patients

Characteristic	Value
Resection	
Yes, n (%)	16 (76)
No, n (%)	4 (19)
Unknown, n (%)	1 (5)
Adjuvant radiation	
Yes, n (%)	5 (24)
No, n (%)	15 (71)
Unknown, n (%)	1 (5)
Adjuvant systemic	
None, n (%)	16 (76)
Immunotherapy, n (%)	4 (19)
BRAF inhibitors, n (%)	1 (5)
Time to recurrence in months mean (alive vs. dead)	48 (50 vs. 12)

Discussion

Modern systemic therapies have improved the treatment of advanced melanoma. Unfortunately, those who progress while on systemic therapy have a poor prognosis. There continues to be an unmet need to augment modern systemic therapy after progression.

This narrative review aims to provide an overview of the clinical characteristics of the reported abscopal effect in melanoma patients as described in the existing literature. However, on screening, we found uncertainties in some of the published melanoma-abscopal literature. For example, resolving non-irradiated lesions were being attributed to the abscopal effect without prior path diagnosis such as regional lymphadenopathy; the authors were not entirely confident that these distal lesions were malignant as opposed to inflammation. Additionally, it was difficult to distinguish between the combined effects of radiation and immunotherapy when applied simultaneously because distal lesion responses could not be entirely attributed to the abscopal effect. To improve the quality of the data, we believe that the definition of the abscopal effect should be carefully and precisely defined and applied in future publications. This will reduce potential confounding issues, allowing easier comparison across reports.

Preclinical radiation abscopal cancer models have been studied and have identified complex immunogenic mechanisms contributing to the abscopal effect. Pulmonary metastasis was decreased in murine models, improving survival when CTLA-4 immunotherapy was combined with radiation [27]. The interdependence of the abscopal effect and the immune system was shown to occur only in immunocompetent mice [28]. Radiation can induce immunogenic cell death, releasing antigens, promoting cytokines, and complement activation, leading to immune responses and subsequent in-situ vaccines [29]. Other postulated mechanisms include promoting expression of major histocompatibility complex class 1, increasing dendritic cell activation and antigen presentation, and ultimately promoting tumor-infiltrating lymphocyte and effector T cell activity to activate systemic immune response after radiation [30].

**Figure 1:**
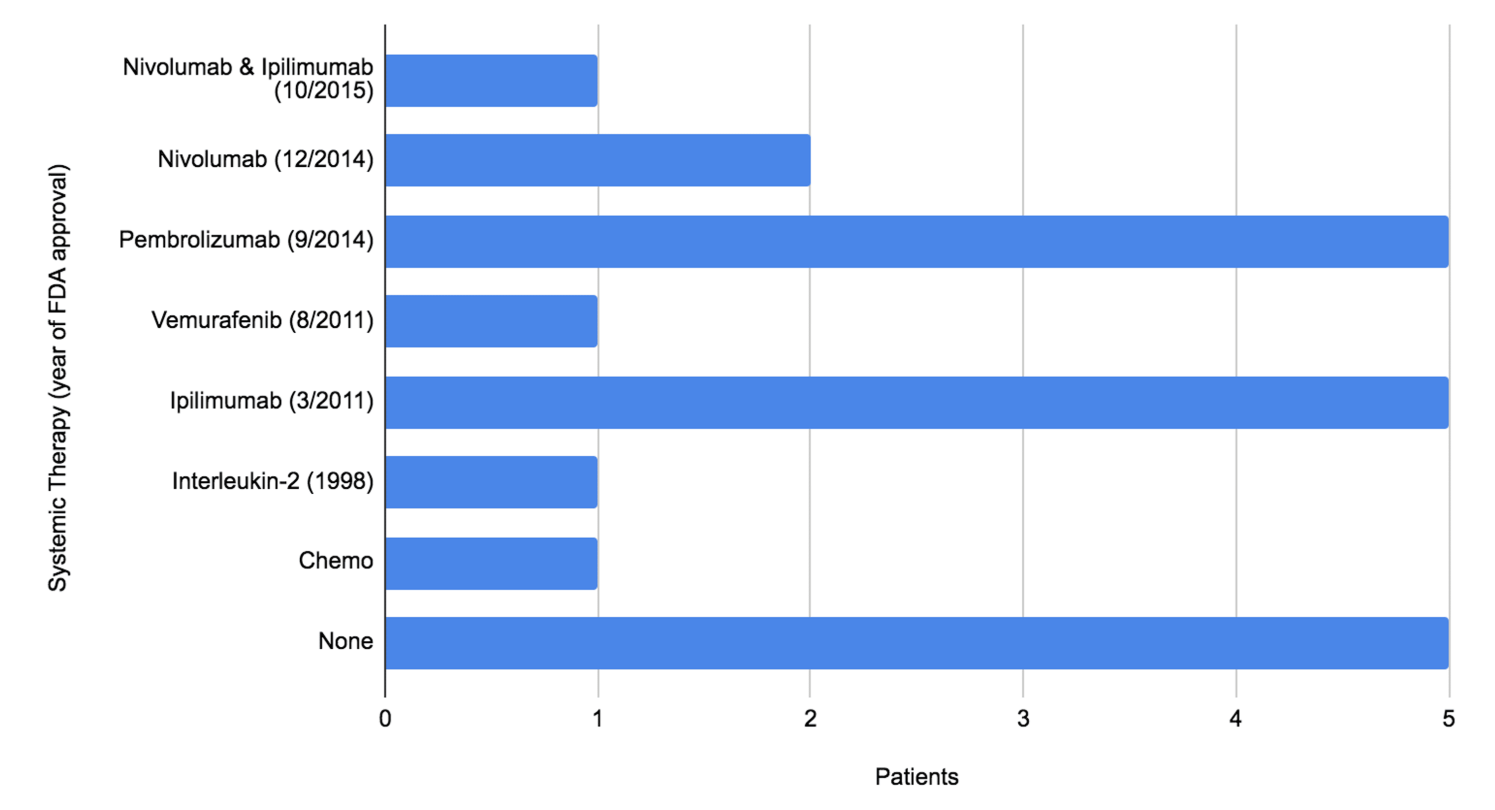
Reported therapies associated with abscopal effect FDA: Food and Drug Administration

The frequency of reported abscopal effects is increased with modern systemic immunotherapy [5]. Our study shows that the abscopal effect occurred in six cases with only radiotherapy and one case of BRAF therapy. The abscopal effect increases to 14 cases (67%) with immunotherapy (interleukin-2, ipilimumab, pembrolizumab, and nivolumab), supporting the underlying role of the immune system in the abscopal effect (Figure 1). This suggests future efforts should continue to focus on optimizing combination radiation-immunotherapy trials and predictors of the abscopal effect.

**Figure 2:**
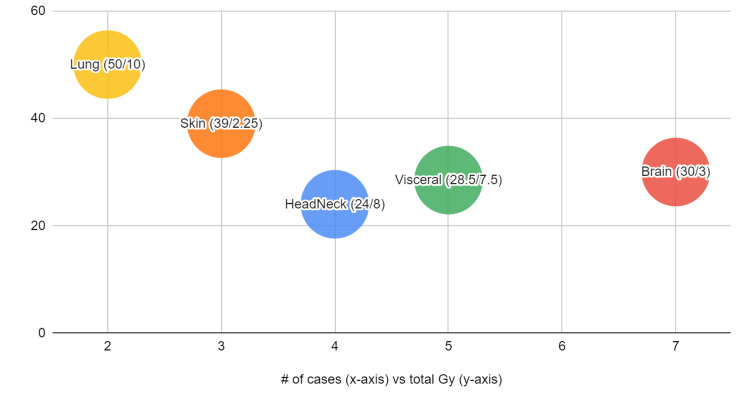
Radiated area (median total/fraction Gy)

Optimal fractionation (hypofractionation vs. conventional fractionation), total dosing, and timing of radiation to induce an abscopal effect remain controversial [31]. Our study observed a mean total Gy of 34 (median total 30 Gy) and mean fractionation Gy of 8 (median fractionation 7.5 Gy). Interestingly, 8 Gy was used in a study involving advanced melanoma treated with radiation and an anti-PDL1 study, which showed a CR and PR rate of 20% and 19%, respectively, and among those with CR, five patients remained disease-free after 9.5 months [16]. This dose of fractionation was also observed in preclinical studies [27,32]. A study showed that three patients who achieved a CR when immunotherapy was combined with radiation in metastatic melanoma were treated with at least 4 Gy but ranged between 4 and 12.5 Gy [33]. Additionally, the mean total Gy to induce abscopal response varied in location as well (Figure 2). Further fractionation studies are needed to understand the best dose for the abscopal effect.

Death from recurrence can still occur even after achieving the abscopal effect. Our study found among the cohort who died, the average age was younger with decreased time to recurrence, decreased overall survival, and decreased abscopal effect duration. Clinically, this suggests that this group warrants ongoing monitoring even after an abscopal effect has been achieved. Among those who died, the mean and median total Gy and fractionation Gy were less (Table 5). The exact mechanism of abscopal effect failure is unclear, but we postulate it is likely due to a reacquired resistance in the interplay between melanoma tumors and the immune system. It’s unclear if checkpoint blockade with radiation at the time of progression after achieving an abscopal effect can restimulate the immune system.

We observed that local responses to radiation therapy could also predict patients more likely to achieve abscopal responses. Of the patients documented to have an abscopal response, 10 had a partial response and 11 had a complete response to local radiation. This was similar to a study that noted that in their cohort of 13 patients with local radiation responses, 11 (85%) patients had an abscopal response and suggested that local responses may predict abscopal response to RT and be a precursor to systemic abscopal response [34]. Further investigations are needed to explore the relationship between irradiated cancer responses and systemic abscopal responses in systemic therapy.

Recently, there has been the publication of a systematic review on melanoma and the abscopal effect [35]. However, the focus was more on radiation and not the accompanying systemic therapy or clinical patient characteristics. We believe our study represents the first study to gather insight into melanoma and abscopal effects involving various systemic therapies along with other pertinent patient clinical data.

Given the extensive literature review, our study provides valuable insights into the abscopal effect in melanoma patients. However, this data should be interpreted with caution given the nature from which these data were derived. The small number of available patients limits the strength of statistical calculations. Heterogeneity in publications resulted in missing values and confounded results as well. Favorable results may relate to patient selection as well. Larger patient cohorts done prospectively are needed to confirm or refute conclusions drawn here. Nonetheless, this provides valuable insight and aids in hypothesis-generating for future efforts at improving the reliability of the abscopal effect.

Prognosis continues to be poor in patients with advanced melanoma progressing after immunotherapy and/or BRAF-targeted therapy. This remains a major unmet need with limited treatment options. The abscopal effect, though rare, may offer hope for these individuals when other treatments have failed.

## Conclusions

Our analysis suggests that providing RT after progression on systemic therapy can result in abscopal responses that appear to be associated with prolonged survival, but death has also been observed, generally in a younger subgroup of patients. There continue to be diverse abscopal responses in various anatomical regions of the body. This adds valuable insight into the role of using palliative radiation as a therapeutic salvage strategy in the treatment of metastatic melanoma patients after progression to modern anti-melanoma systemic therapy. Well-designed clinical trials and refining abscopal definitions are needed to confirm these retrospective observations.
